# Purification of Human Immunoglobulin G with Bathophenanthroline–Zn^2+^, –Fe^2+^, or –Cu^2+^ Complexes

**DOI:** 10.3390/antib14020040

**Published:** 2025-05-12

**Authors:** Thisara Jayawickrama Withanage, Ron Alcalay, Olga Krichevsky, Ellen Wachtel, Ohad Mazor, Guy Patchornik

**Affiliations:** 1 Department of Chemical Sciences, Ariel University, Ariel 70400, Israel; thisarawazz@gmail.com (T.J.W.);; 2 Israel Institute for Biological Research, Ness Ziona 7410001, Israel; 3 Faculty of Chemistry, Weizmann Institute of Science, Rehovot 76100, Israel

**Keywords:** antibody purification, IgG, non-chromatographic, ligand-free, Protein A

## Abstract

Background/Objectives: Pharmaceutical companies are aware of the ongoing effort to satisfy the increasing global demand for therapeutic-grade monoclonal antibodies (mAbs), an especially difficult challenge for poor and developing countries. We present a simple, economical, single-step purification approach at neutral pH for polyclonal human IgG (hIgG), which does not require any expensive ligands, chromatography columns, polymers, or membranes. Methods/Results: Instead, porous precipitates of commercial, recyclable aromatic [bathophenanthroline:cation] complexes were found to efficiently capture impurity proteins from CHO cells or *E. coli* lysate while maintaining the majority of the highly concentrated hIgG (5–15 mg/mL) in the supernatant. [(Batho)_3_:Zn^2+^] complexes were the most promising, resulting in hIgG with a purity of ≈95%, by SDS-PAGE. This purified hIgG is monomeric (by dynamic light scattering, DLS) and preserves the native secondary structure (by far UV circular dichroism spectroscopy, CD). The process yield is >90% (by densitometry) and is maintained after a 100-fold increase in the reaction volume, which required only proportional increases in reagents. Conclusions: Although Protein A chromatographic columns, the industry gold standard, have a limited binding capacity, are costly, and require familiarity with column maintenance, we are attempting, by our efforts, to help to produce a more efficient, simple, and economical purification platform.

## 1. Introduction

Antibodies are increasingly being exploited as biological pharmaceuticals, as well as diagnostic tools [1,2,3]. The majority of the antibodies currently in clinical use belong to the immunoglobulin G isotype (IgG), and their contributions to global health are best reflected in the increasing number of new therapies that are antibody based [4,5]. The world-wide consumption of hundreds of kilograms of specific monoclonal antibodies per year explains the growing need for improving the efficiency at which antibodies are manufactured at an industrial scale [6]. Accordingly, intense effort has been directed, during the past few decades, toward increasing the expression levels of IgG antibodies (i.e., upstream processing). This objective has required the optimizations of cell lines, media, and bioreactor conditions and has been translated into high-concentration cell culture IgG titers that may reach 5–13 g/L [7,8,9,10] and even 25 g/L [11,12].

However, the high antibody concentration in cell culture broths poses a major challenge for antibody purification. This challenge derives from the need to capture the majority of the IgG population within a single chromatographic step, while the antibody concentration may, in fact, exceed the binding capacity of the particular chromatographic column in use. Moreover, at high IgG titer concentrations, clarification techniques responsible for preventing large particles, cell debris, and whole-cell impurities from entering the column become more difficult and, accordingly, the cost of the antibody production is increased [13]. These challenges and the unavoidable requirement for one or two additional polishing steps needed to exclude residual amounts of impurities (e.g., host cell proteins, DNA, and viruses [14]), are estimated to represent ~50% of the overall production costs [15] and make IgG downstream processing the bottleneck for industrial-scale production [10,16].

IgGs are generally purified via column chromatography and a ligand called Protein A. Protein A is a 42 KDa *Staphylococcus aureus* bacterial protein [17] that binds to the crystallizable (Fc) domain of diverse IgGs with high affinity (K_d_~2 × 10^−9^ M) [18] and specificity [19], thereby excluding viruses, host cell proteins (HCPs), and DNA [20,21,22]. The removal of these impurities is efficient, as the generally observed levels of HCPs are <100 ppm and those of host cell DNA are <10 pico/gr/dose [23]. These unique properties of Protein A translate into high process yields (≥95%) and purity levels (>98%) within a single chromatographic step [24,25] and explain why Protein A chromatography has become the gold-standard technology in antibody purification. However, being a chromatographic technique, it relies on immobilizing the ligand on polymeric resins, which, in turn, introduces several technical challenges (e.g., ligand leaching [14,24], ligand deamidation [26], and acidic-pH-induced antibody aggregation [27,28,29]). Perhaps the current major challenge for biotechnology is to find a practical avenue in which industrial-scale Protein A columns will capture the majority of the antibodies present in highly concentrated bioreactors [7,8,9,10]. Such an objective requires the development of Protein A resins with (a) high binding capacities, (b) high flow rates, as well as (c) stability after multiple reuse cycles (100–200) and would not be affected significantly during column cleaning and sanitation (conducted at high pH levels, e.g., in 0.1–0.5 M NaOH) at the end of each purification cycle and still reach process cost effectiveness [30]. In this regard, we note that several non-chromatographic strategies for antibody purification have been studied with the intention of replacing Protein A columns, but, to the best of our knowledge, none of these technologies is considered to be a practical alternative to Protein A chromatography [31,32,33,34,35,36].

To address the above challenges, we recently described a non-chromatographic, ligand-free antibody purification approach exploiting non-ionic surfactant micelles specifically conjugated to each other through [metal:chelator] amphiphilic complexes [37,38,39,40]. These conjugated micellar aggregates efficiently purified polyclonal human IgG (hIgG) at concentrations of 15–25 mg/mL [37] in two sequential steps: HIgG was first captured by the conjugated micelles in the presence of osmotically active PEG6000 (while more hydrophilic impurities were rejected) and were then extracted from the micelles without the parallel coextraction of impurities [37]. Thus, purification required the initial binding of hIgG to the micellar aggregates. In the current study, we have sought to simplify the general protocol and have, therefore, removed the surfactant and polyethylene glycol (PEG) components. Hence, purification cannot rely on antibody binding to conjugated micelles in the presence of an osmotically active polymer (e.g., PEG). Consequently, in this report, we assess the possibility of separating the artificial background impurities *E. coli* lysate proteins or CHO-cell-extruded proteinsfrom concentrated hIgG samples (15 mg/mL) with the use of precipitated [metal:chelator] complexes that serve as the “purification tool”. This purification tool is composed of the aromatic chelatorbathophenanthroline (batho)bound to either Zn^2+^, Fe^2+^, or Cu^2+^.

**Figure 1:**
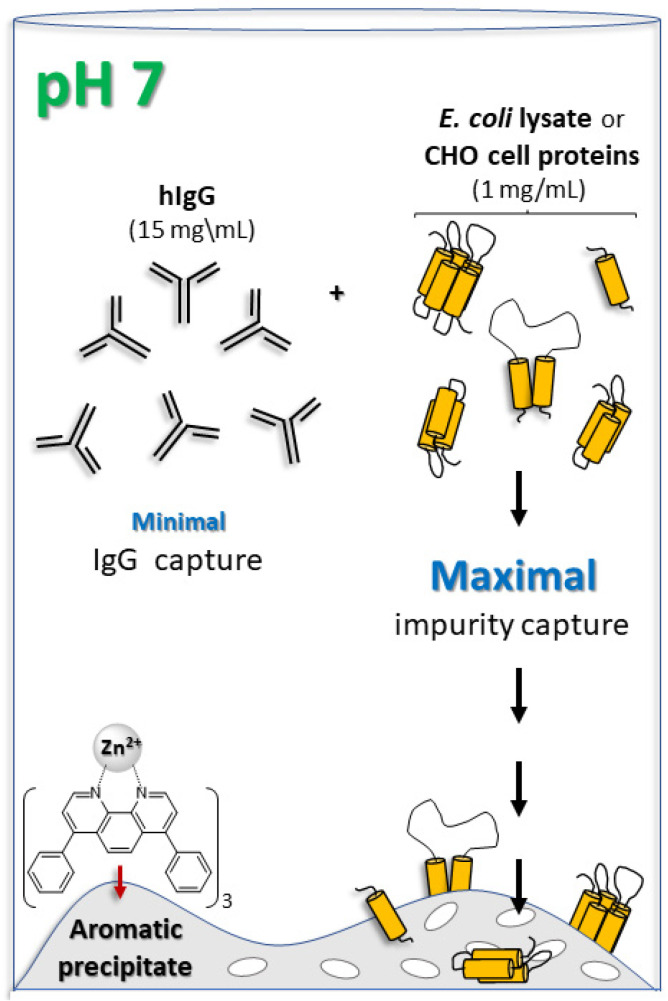
Schematic illustration of the purification protocol for concentrated (15 mg/mL) human IgG (hIgG). A 30 min incubation in 50 mM NaPi, pH 7 at 10 °C for polyclonal hIgG plus *E. coli* lysate or proteins secreted from CHO cells (1 mg/mL) and mixed with the washed, porous, aromatic precipitate leads to the capture of the majority of the protein impurities. These impurities serve to mimic the expression of the cells’ proteinaceous content. Most of the hIgG molecules remain in the supernatant. The schematic illustration is not drawn to scale.

We have recently reported that human lactoferrin, which is highly positively charged, binds efficiently to precipitated [(batho)_3_:Fe^2+^] fully aromatic complexes and have suggested that binding occurs via [cation:π] interactions [41]. Similar efficient binding to the aromatic complex precipitate was observed for an Fc-fusion protein, where the protein involved was acetylcholinesterase, and the origin of the Fc moiety was IgG1 [42]. Acetylcholinesterase presents a strongly negative surface charge [43], which would not be conducive to interaction with the *π*-cloud of the aromatic complex. Rather, the more likely active player in binding was thought to be the Fc component of the fusion protein [44]. We were, therefore, motivated to investigate whether the precipitated [(batho)_3_:Fe^2+^] aromatic complex might be used to efficiently separate intact polyclonal hIgG from mixtures with manually added protein impurities. If so, such an optimized protocol could expedite and simplify antibody production in a cost-effective manner. A schematic illustration of the proposed strategy is shown in Figure 1.

## 2. Experimental

### 2.1. Materials

Sodium chloride (Sigma, S7653), bathophenanthroline (GFS chemicals, C038446), zinc chloride (Sigma, 208086), copper(II) sulfate pentahydrate (Sigma, 209198), FeCl_2_ (Sigma, 450936), and a Protein A HP Spin-Trap^TM^ column (GE, 2809031-32) were used. *E. coli* lysate was provided by the Israel Structural Proteomics Center (Weizmann Institute of Science), impurity proteins from Chinese hamster ovary (CHO) cells were provided by the Institute of Biological Research (IIBR), and human polyclonal IgG (Lee Biosciences, 340–21, 95% pure) was used. PEG 6000 (Sigma Aldrich, Bellefonte, PA, USA, average MW 6000, cat # 8.07491) was used. All the reagents were analytical grade.

### 2.2. Methods

#### 2.2.1. Preparation of 200 mM Bathophenanthroline:DMSO:HCl Solution

To 90 μL of dimethyl sulfoxide (DMSO) and 10 μL of 25% HCl, 6.64 mg of bathophenanthroline was added, and the mixture was vortexed for 5 min at 25 °C until total dissolution was observed.

#### 2.2.2. Polyclonal IgG Purification—Step I: Preparation of [(Batho)_x_:Cation_y_] Complexes

Each of the three complexes was generated by mixing equal volumes of media A and B as follows: Medium A was prepared by the addition of 1 μL of the amphiphilic chelator bathophenanthroline to 25 μL of medium B, comprising 4 mM ZnCl_2_ or 4 mM CuSO_4_ 5H_2_O or 4 mM FeCl_2_ in 20 mM NaCl. The mixture was vigorously vortexed, incubated for 5 min at 25 °C, followed by the addition of 3.5 μL of 5 M NaCl. After an additional 5 min incubation at 25 °C, centrifugation was applied (at 21,000× *g* for 5 min at 19 °C), and the supernatant was removed. The resulting pellet was washed with 50 μL of cold 20 mM NaCl. Centrifugation followed (at 21,000× *g* for 5 min at 10 °C).

#### 2.2.3. Polyclonal IgG Purification—Step II: Impurity Capture

Each of the freshly prepared [bathophenanthroline:cation] complex precipitates was resuspended in 100 μL of a mixture of 15 mg/mL polyclonal human IgG, 50 mM sodium phosphate buffer (pH 7.0), and either 0.6 mg/mL of *E. coli* cell lysate or 0.16 mg/mL of CHO-cell-excreted proteins. The suspension was vigorously vortexed for 2 min and incubated at 10 °C for 30 min. A short spin was applied (at 21,000× *g* for 5 min at 10 °C), and the supernatant composition was analyzed using SDS-PAGE. PEG 6000 was not present.

#### 2.2.4. SDS-PAGE Electrophoresis

SDS-PAGE was run on a 10% polyacrylamide gel in the presence of a reducing agent (β-mercaptoethanol.)

#### 2.2.5. Binding Capacity of the [(Batho)_3_:Zn^2+^] Complex for Impurity Proteins

Freshly prepared [(batho)_3_:Zn^2+^] complex precipitates were resuspended in 100 μL of XXX containing 0.25, 0.5, 0.75, or 1 mg/mL of *E. coli* cell lysate or 1 mg/mL of CHO-cell-excreted proteins. Following vigorous vortexing for 2 min, the suspension was incubated at 10 °C for 30 min. A brief spin followed (at 21,000× *g* for 5 min at 10 °C), and the supernatant was analyzed using SDS-PAGE.

#### 2.2.6. Binding Capacity of the [(Batho)_3_:Zn^2+^] Complex, Mw 1061 Daltons, for Impurity Proteins

Freshly prepared [(batho)_3_:Zn^2+^] complex precipitates were resuspended in 100 μL of 0.25, 0.5, 0.75, or 1 mg/mL of *E. coli* cell lysate or 1 mg/mL of CHO-cell-excreted proteins. Following vigorous vortexing for 2 min, the suspension was incubated at 10 °C for 30 min. A brief spin followed (at 21,000× *g* for 5 min at 10 °C), and the supernatant was analyzed using SDS-PAGE.

#### 2.2.7. Bradford Assay of Impurity Proteins

A Bradford assay was used to prepare a calibration curve for the protein concentration based on bovine serum albumin (BSA). The assay was run via serial dilutions of BSA in DDW (e.g., 0.1, 0.2, 0.4, 0.6, 0.8, and 1.0 mg/mL). Samples (50 µL) of either *E. coli* lysate or CHO-cell excreted proteins were mixed with 1 mL of the “Bradford reagent” containing 10 mg of Coomassie brilliant blue G-250, 5 mL of ethanol, 1 mL of 32% hydrochloric acid, and 94 mL of deionized water and incubated for 5 min. The optical density (OD) was measured at 595 nm on a Jasco V-750 (Jasco, Tokyo, Japan) spectrophotometer and compared to the BSA calibration curve.

#### 2.2.8. Scanning Electron Microscopy Imaging

Freshly prepared [(batho)_3_:Zn^2+^], [(batho)_3_:Fe^2+^], and [(batho)_2_:Cu^2+^] complexes were precipitated as described above. The resulting pellets were resuspended in 50 µL of DDW, and samples for SEM were prepared by dipping a grid into the suspension and drying the grid overnight at 25 °C in a vacuum desiccator. Images of the zinc and copper complexes were obtained using the UHR-MAIA3 TESCAN scanning electron microscope with In-Beam SE and STEM Bright detectors at HV 25 kV and magnification X 400K. For the iron complex, the lower resolution analysis mode at HV 5 kV and magnification X 40K was used. This change was necessitated because of the strongly ferromagnetic properties of Fe^2+^, which could damage or contaminate the electromagnetic coil element of the microscope and/or the detector.

#### 2.2.9. Dynamic Light Scattering (DLS)

Polyclonal hIgG, purified via each of the three [metal:chelator] complexes studied, was compared to hIgG purified in a Protein A chromatographic column (see Section 2.2.9) and to untreated commercial (purity ≥ 95%) hIgG. Samples were diluted with phosphate-buffered saline (PBS) to 0.5 mg/mL (pH 7.4) and centrifuged at 21,000× *g* for 10 min at 10 °C prior to the analysis. The intensity-weighted particle size distribution of the hIgG was determined using the auto correlation spectroscopy protocol of the Nanophox instrument (Sympatec GmbH, Clausthal–Zellerfeld, Germany).

#### 2.2.10. Circular Dichroism (CD) Spectroscopy

Polyclonal hIgG, purified via each of the three [metal:chelator] complexes studied, was compared to hIgG purified via a Protein A chromatographic column and to untreated commercial (purity ≥ 95%) hIgG. All the samples were diluted with 50 mM sodium phosphate (pH 7) to 0.05 mg/mL and analyzed with a Chirascan CD spectrometer (Applied Photophysics, Charlotte, North Carolina, USA). CD spectra report ellipticity (θ), which is proportional to the difference in the absorbances of left and right circularly polarized light [θ = 3300° (A_L_ − A_R_)], as a function of the wavelength. A quartz cell, with a path length of 0.1 cm, was used for the measurements. The CD spectra were recorded with a 1 nm bandwidth resolution in 1 nm steps at 20 °C and corrected for baseline distortion by subtracting the reference spectrum of the corresponding buffer solution.

#### 2.2.11. HIgG Purification in a Protein A HP Spin-Trap^TM^ Column

Polyclonal hIgG, in the background of CHO-cell impurity proteins, was purified in an HP Spin-Trap^TM^ column. The mixture (400 µL) contained 64 µL of CHO-cell impurity proteins (0.5–1 mg/mL) and hIgG at a final concentration of either 5 or 15 mg/mL in 20 mM sodium phosphate buffer (pH 7.0). After 4 min of incubation at 25 °C with gentle mixing, centrifugation (30 s at 100× *g*) was applied. The Protein A column was then washed twice with 400 µL of the binding buffer supplied by the column’s manufacturer. Following a further 4 min of incubation, hIgG was eluted in the presence of 100 mM Gly (pH 2.7) at 25 °C with gentle mixing. The elution step was repeated twice. The elution fractions were then combined and centrifuged for 30 s at 10 °C at 100× *g* prior to SDS-PAGE gel loading.

## 3. Results and Discussion

Three different [chelator:divalent cation] complexes were investigated, i.e., [(batho)_3_:Fe^2+^] [45], [(batho)_3_:Zn^2+^] [46], and [(batho)_2_:Cu^2+^] [47].

### 3.1. Complex Precipitate Morphologies

**Figure 2:**
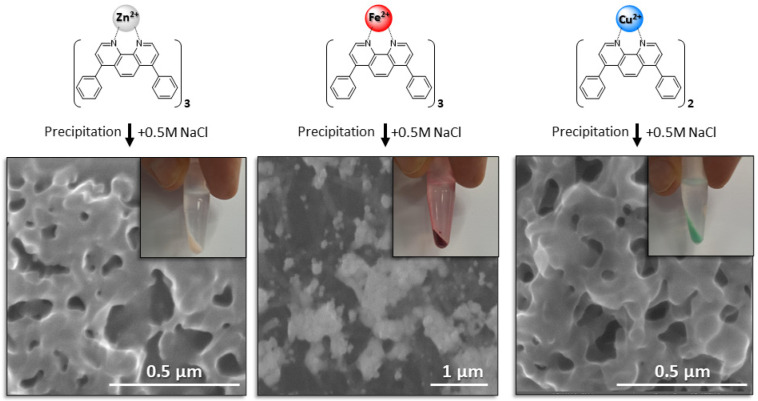
The bathophenanthroline:divalent cation complexes form a colored (see inset photographs), micro-porous (see SEM imaging) precipitate in 0.5 M NaCl.

Each of the three complexes was precipitated in a high-concentration salt solution (0.5 M NaCl) prior to incubation with a mixture of polyclonal hIgG and cellular impurity proteins. Scanning electron microscopy (SEM) imaging revealed that the [(batho)_3_:Zn^2+^], [(batho)_3_:Fe^2+^], and [(batho)_2_:Cu^2+^] complex precipitates are highly porous, non-crystalline materials with a broad distribution of pore sizes, on the order of several hundred nanometers (Figure 2). The decision to use the commercially available batho chelator was motivated by its extremely low water solubility, which (1) allowed quantitative recycling by recrystallization and (2) minimized leaching into the purified hIgG.

### 3.2. Comparison of Process Yields for Different Divalent Cations

**Figure 3:**
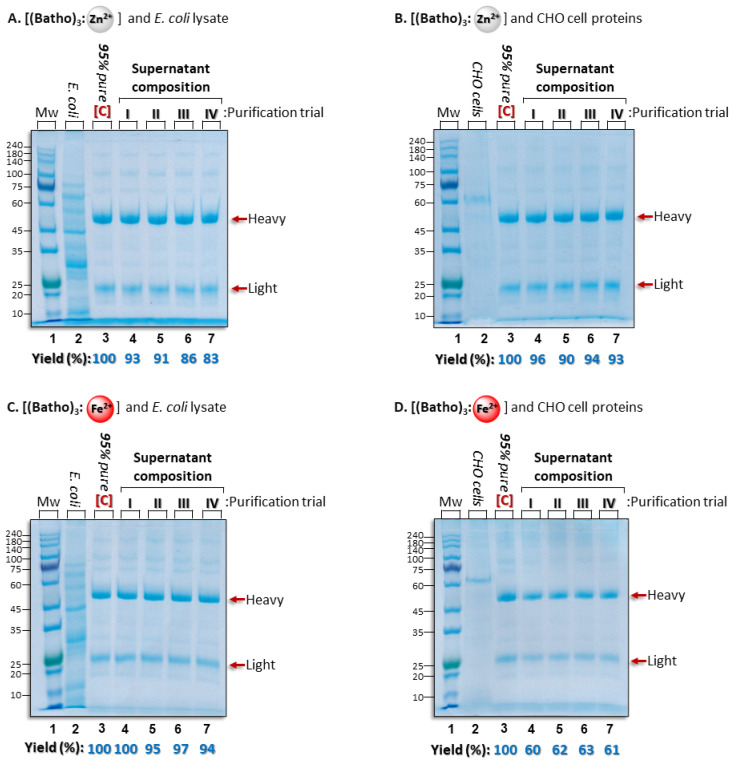

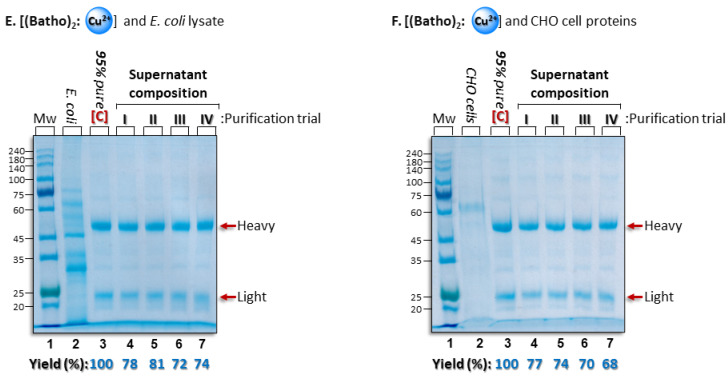
SDS polyacrylamide gels (with beta-mercaptoethanol) reveal hIgG purification process efficiency using zinc (**A**,**B**), iron (**C**,**D**), or copper (**E**,**F**) divalent cations in the presence of the relevant impurity protein background. Lane 1: molecular weight markers; lane 2: the total amount added of the artificial contamination background; lane 3: the total amount of the commercial polyclonal hIgG (≥95% purity by HPLC) ([C]) added to each of the purification trials; lanes 4–7: recovered hIgG after incubation for 30 min at 10 °C in 50 mM NaPi (pH 7). Each lane represents an independent purification trial performed on a different day. Process yields were determined by comparing the intensities of the bands representing the total amount of the commercial hIgG ([C]) added to each purification trial (lane 3) to that of the hIgG present in the supernatant after the impurity protein removal. Quantitation is performed using ImageJ, v.1.54p (NIH). Red arrows labelled “Heavy” or “Light” show the migration of the reduced heavy and light chains of the hIgG population. Gels are Coomassie stained.

Although recombinant monoclonal antibodies (mAbs) for therapeutic use are commonly produced in Chinese hamster ovarian cells (CHO cells) [48,49], expression in *E. coli* is currently becoming a realistic alternative [50]. The efficiency of the impurity removal from both of these cell-derived backgrounds was, therefore, tested by performing “spike experiments”, in which a known amount of the target protein is mixed with contaminants; the process yield and purity are then quantitated using SDS-PAGE gel electrophoresis (Figure 3A–F). The concentration of the hIgG was maintained at 15 mg/mL, a higher antibody titer than commonly observed (<10 mg/mL) [51]. Under these working conditions, we found that all three aromatic complexes, independent of the cation used, removed protein impurities excreted by CHO cells, as well as those that derive from the lysate of *E. coli* cells. This led to an IgG purity level similar to that of the commercial control (~95% purity by HPLC) (Figure 3A–F, lane 3). However, the process yield was clearly dependent on the cation used. The highest yield from both contamination backgrounds was observed with the [(batho)_3_:Zn^2+^] complex (avgs. of 88% and 94% in *E. coli* and CHO cells, respectively), and the lowest efficiency was with the [(batho)_2_:Cu^2+^] complex (avgs. of 76% and 72% in *E. coli* and CHO cells, respectively) (Figure 3A,B,E,F). The [(batho)_3_:Fe^2+^] complex exhibited a different behavior. It was highly efficient in the presence of bacterial lysate proteins but performed poorly with excreted CHO-cell proteins (avgs. of 96% and 61% in *E. coli* and CHO cells, respectively) (Figure 3C,D). The reason for this is still not clear. One possible, if partial, explanation is the absence of complex post-translational modifications, such as *N*-linked glycosylation in the CH2 domain of the Fc moiety, in *E. coli*. To produce complex biologics, such as mAbs, mammalian cell lines became the hosts of choice despite obvious problems because of glycan heterogeneity, lengthy and costly production processes, and the need for viral clearance. Glycosylation was thought to be necessary for the improved biophysical properties and serum stability of antibodies in addition to their Fc-mediated effector functions. However, a comparison of glycosylated versus non-glycosylated antibodies, produced either in mammalian cell lines or in *E. coli*, demonstrated nearly identical properties in vitro and in vivo, including a serum half-life (t_1/2_), except for effector functions [21,30,31]. In the near future, non-glycosylated antibodies could become the default format of therapeutics, where various effector functions are either unnecessary or detrimental. With all the complexes, process reproducibility was demonstrated by repeating the purification protocol at least four times on different days (Figure 3A–F, lanes 4–7).

### 3.3. Binding Capacity of [(Batho)_3_:Zn^2+^] Aromatic Complexes for Protein Impurities

The optimal amount of aromatic complexes for the preferential binding of protein impurities (Figure S1) was found to be a critical parameter. Focusing on the more consistently efficient [(batho)_3_:Zn^2+^] complex (cf. Section 3.2), protein impurities derived from either CHO cells or *E. coli* are captured with 66 μmol of [(batho)_3_:Zn^2+^]. The strict adjustment of the hIgG concentration, as well as the absence of osmotically active PEG6000 (Figure S2A,B), was also required. Ideal working conditions would favor the maximal impurity binding in parallel with the minimal hIgG capture: HIgG would, therefore, remain in the supernatant. We found that limiting the hIgG concentration to 5–15 mg/mL, along with the impurity concentration to 0.5–1 mg/mL, results in the optimal molar ratio of the IgG:batho complex, 0.0033–0.01 mmol:0.0002 mmol.

### 3.4. Optimizing pH and Buffer Concentration for the HIgG Purification Protocol

We surveyed possible buffers and pH values, which would maximize the yield and purity of the hIgG purification protocol. We found that Na citrate, which can be titrated to three different pK values, allowed us to determine that a neutral pH was optimal (Figure S3 and Figure S4). Once 50 mM Na citrate, pH 7, suggested the optimal working conditions, other buffers (Tris and NaPi) were assessed at the same pH (Figure S5). The advantage of NaPi relative to DDW was clearly observed and appeared to have the maximum positive effect at 50 mM NaPi. Increasing the buffer concentration to 100 mM (Figure S5A, lanes 4–5) did not produce any improvement. A concentration of 50 mM NaPi (pH 7) was, therefore, chosen as the best concentration for this buffer. Similar behavior was found with Tris buffer, reaching the maximal impact at 50 mM Tris (pH 7) (Figure S5B, lane 4). Nevertheless, NaPi remained the buffer of choice as being more biologically relevant.

### 3.5. Native, Non-Aggregated State of HIgG

**Figure 4:**
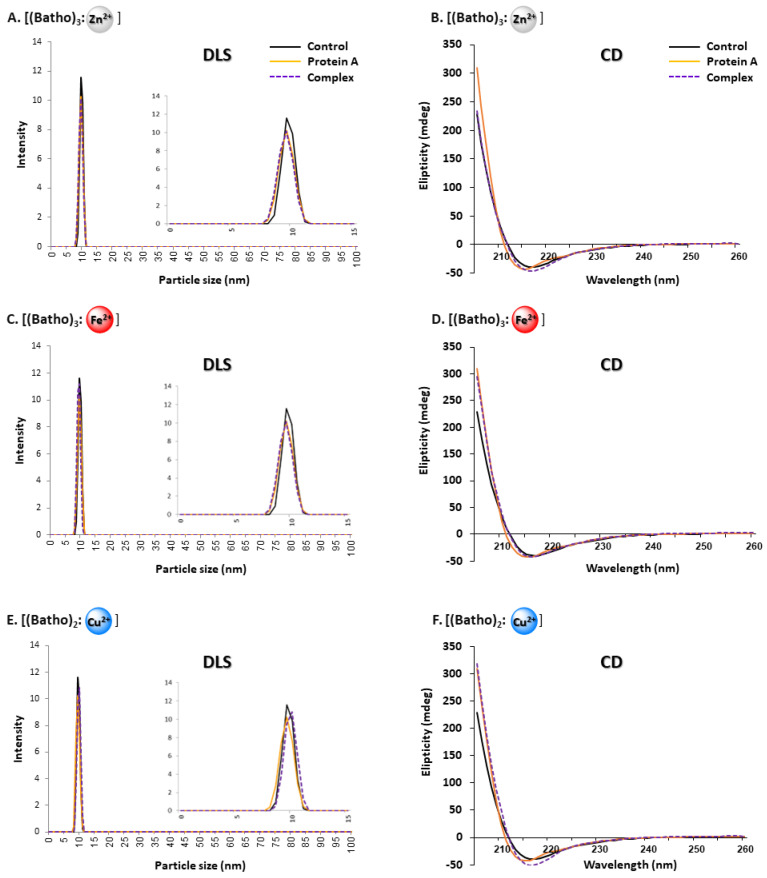
(**A**,**C**,**E**): Hydrodynamic particle size distributions, as determined using dynamic light scattering (DLS), of purified polyclonal hIgG diluted with PBS (pH 7.4) to 0.5 mg/mL and analyzed at 25 °C. Legend: purified with the aromatic complex (dotted purple line); purified in a Protein A column (red/orange line); or commercial, as-received hIgG (purity by HPLC, 95%) that had not been exposed to any additional purification protocol (black line). (**B**,**D**,**F**): Protein secondary structure as determined using far-UV circular dichroism (CD) spectroscopy. Sample identification as in panels (**A**,**C**,**E**) but at an hIgG concentration of 0.05 mg/mL in 50 mM NaPi at 25 °C.

The aggregational state of the hIgG remaining in the supernatant following impurity capture by the precipitated complexes was studied with dynamic light scattering (DLS) (Figure 4A,C,E, left-hand side). Single peaks, representing particle sizes ranging between 9 and 11 nm, were observed for all three aromatic chelators. These values were in agreement with those of the control sample, in which 95% pure commercial hIgG was not subjected to any additional purification, as well as the hIgG that was purified with a Protein A column (see Section 3.6). This range of diameters is also consistent with those in our previous studies [36,40,52]. Because no additional peaks were observed, we conclude that the purified hIgG is monomeric. This finding may not be surprising when considering the fact that the conditions were designed to suppress hIgG binding to the aromatic complexes. Thus, the postulated minimal interaction between hIgG and the aromatic complexes should not significantly modify the antibody secondary structure. This question was also studied with circular dichroism (CD) spectroscopy, a powerful, non-invasive, and quantitative tool to assess structural alterations in antibodies, in particular [53], and proteins, in general [54], under different environmental conditions.

### 3.6. Comparison of Aromatic Complex Purification and Protein A Chromatography

**Figure 5:**
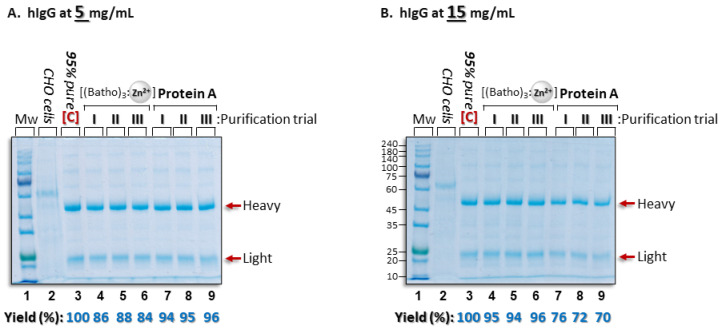
SDS polyacrylamide gels with beta-mercaptoethanol allow the comparison of the polyclonal hIgG purification efficiencies obtained via the [(batho)_3_:Zn^2+^] complex (66 µM) in 50 mM Na citrate (pH 7) or with a Protein A column at antibody concentrations of either 5 mg/mL (**A**) or 15 mg/mL (**B**). (**A**) Lane 1: Molecular weight markers; lane 2: the total amount of the CHO-cell-extruded impurity proteins (1 mg/mL) to be mixed with 5 mg/mL of commercial polyclonal hIgG ([C]); lane 3) and added to each of the purification trials; lanes 4–6 and 7–9: Recovered hIgG via the [(batho)_3_:Zn^2+^] complex or eluted from a Protein A column (as described in Section 2.2). (**B**) As in (**A**) but at a concentration of 15 mg/mL of hIgG. Three independent purification trials were conducted on different days. Gels are Coomassie stained. Process yields were determined by comparing the intensities of the bands representing the total amount of the commercial hIgG ([C]) added to each purification trial (lane 3) to the intensities of the bands following each purification trial.

We compared the [(batho)_3_:Zn^2+^] purification capability with that of a Protein A column (Figure 5). The process performance was evaluated at two hIgG concentrations: 5 mg/mL and 15 mg/mL. The 5 mg/mL sample presents ideal conditions for the Protein A column, and, indeed, the recovery yields of the hIgG were very high (94–96% by densitometry) (Figure 5A, lanes 7–9) while the [(batho)_3_:Zn^2+^] protocol, calibrated for 15 mg/mL IgG, led to lower efficiency (84–88% by densitometry) (Figure 5A, lanes 4–6). We attribute this to the presence of excess aromatic complexes relative to the number of hIgG antibodies in the system. However, at 15 mg/mL of IgG, where the [(batho)_3_:Zn^2+^] protocol had been optimized, the process efficiency improved to 94–96% yield (Figure 5B, lanes 4–6), while that of the Protein A column was in the 70% range (Figure 5B, lanes 6–9). The latter result was not unexpected; the concentration of antibodies exceeded the binding capacity of the Protein A column. As far as the purity was concerned, we found that the Protein A column led to slightly purer hIgG relative to that achieved with the [(batho)_3_:Zn^2+^] complex (Figure 5A,B, lanes 4–6 vs. 7–9). This difference can be understood when considering the fact that Protein A is a highly specific ligand for the Fc moiety of IgG, whereas our protocol is based on non-specific interactions. These encouraging findings (i.e., high yield and purity rates) with concentrated antibody samples (15 mg/mL) imply that commonly employed Protein A column chromatography could be replaced by a non-chromatographic, ligand-free step, thereby reducing both cost and time for antibody downstream processing.

### 3.7. Increasing the Reaction Volume

To represent a reasonable starting point for large-scale production, it was essential to demonstrate process efficiency with larger purification volumes. The basic laboratory protocol, applied at the 0.1 mL scale, was increased 10-, 25-, 50-, and 100-fold. We observed that only proportional reagent adjustments were required in order to maintain high levels of the process yield and purity (Table 1). Although 10 mL is far from the production scale (5000–25,000 L) used by most biopharmaceutical companies [55], these results are, nevertheless, encouraging.

## 4. Conclusions

We have presented a simple, economical, two-step purification procedure at a neutral pH for polyclonal human IgG (hIgG) antibody molecules, which does not require any expensive ligands, chromatography columns, polymers, or membranes. Instead, the active media consist of porous precipitates of commercial, recyclable, aromatic [bathophenanthroline:cation] complexes. The [(batho)_3_:Zn^2+^] complexes were found to efficiently capture manually introduced impurity proteins (at concentrations of ≤1 mg/mL) from CHO cells or *E. coli* lysate, while the majority of the hIgG (at concentrations of 5–15 mg/mL) antibody molecules remained in the supernatant. The fact that process efficiency was observed at such high IgG concentrations (15 mg/mL) implies that our purification platform can, indeed, serve current, and perhaps even future, high-concentration upstream IgG titers. We note, however, that this approach is necessarily limited to cell culture working conditions in which the concentration of the target antibody exceeds that of the impurity protein background by more than a factor of 10 and that of the chelator by at least a factor of 15.

## Figures and Tables

**Table 1:** Purification process upscaling. The purification protocol for 15 mg/mL of hIgG via the [(batho)_3_:Zn^2+^] complex at 66 μmol and in the presence of 1 mg/mL of CHO-cell-secreted proteins was applied to increasing reaction volumes while raising proportionally the amounts of all the required reagents. The mass ratio of the hIgG: the total amount of CHO impurity proteins and the mass ratio of the [(batho)_3_:Zn^2+^] complex: the total amount of CHO impurity proteins remain unchanged. The buffer was 50 mM NaPi buffer, pH 7. Standard deviations, based on triplicate measurements, ranged from 1.5 to 4%.

Reaction Volume	0.1 mL	1 mL	2.5 mL	5 mL	10 mL
Yield (%)	93 ± 4	93 ± 4	92 ± 4	90 ± 4	94 ± 4
Purity (%)	95	94	95	95	94

## Data Availability

The data presented in this study are available on request from the corresponding authors.

## Supplementary Materials

The following supporting information can be downloaded at https://www.mdpi.com/article/10.3390/antib14020040/s1, Figure S1: SDS-PAGE electrophoresis (with β-mercaptoethanol) displaying background impurity proteins present in the supernatant with (+) or without (−) the [(batho)_3_:Zn^2+^] complex; Figure S2: SDS-PAGE electrophoresis (with β-mercaptoethanol) of the supernatant reveals increasing capture of hIgG by the [(batho)_3_:Zn^2+^] complex as a function of the PEG-6000 concentration; Figure S3: SDS-PAGE analysis of the supernatant under reducing conditions in the presence of hIgG (15 mg/mL), *E. coli* lysate (1 mg/mL), 66 µmole of the [(batho)_3_:Zn^2+^] complex in 50 mM Na citrate buffer at the pH values indicated; Figure S4: SDS-PAGE analysis of the supernatant, run under reducing conditions, in the presence of CHO cell impurity proteins (1 mg/mL), 66 µmole of the [(batho)_3_:Zn^2+^] complex; buffer concentration at pH7, as indicated.
