# Supplementary material for: Purification of Human Immunoglobulin G with Bathophenanthroline–Zn2+, –Fe2+, or –Cu2+ Complexes
Source: Antibodies (Basel). 2025 May 12;14(2):40. doi: 10.3390/antib14020040 (PMC12101337; doi:10.3390/antib14020040)
Supplement: Supplementary file 1 [file antibodies-14-00040-s001.zip › antibodies-3561248-supplementary.pdf]

Purification of human IgG with bathophenanthroline Zn^{2+}, Fe^{2+} or Cu^{2+} complexes

Supplementary

Thisara Jayawickrama Withanage,[1] Ron Alcalay [2] Olga Krichevsky [1] Ellen Wachtel [3] Ohad Mazor [2*] and Guy Patchornik [1*]

[1] Department of Chemical Sciences, Ariel University, 70400, Ariel, Israel.

[2] Israel Institute for Biological Research, Ness Ziona, 7410001, Israel.

[3] Faculty of Chemistry, Weizmann Institute of Science, 76100, Rehovot, Israel.

*Corresponding authors emails:

guyp@ariel.ac.il

ohadm@iibr.gov.il

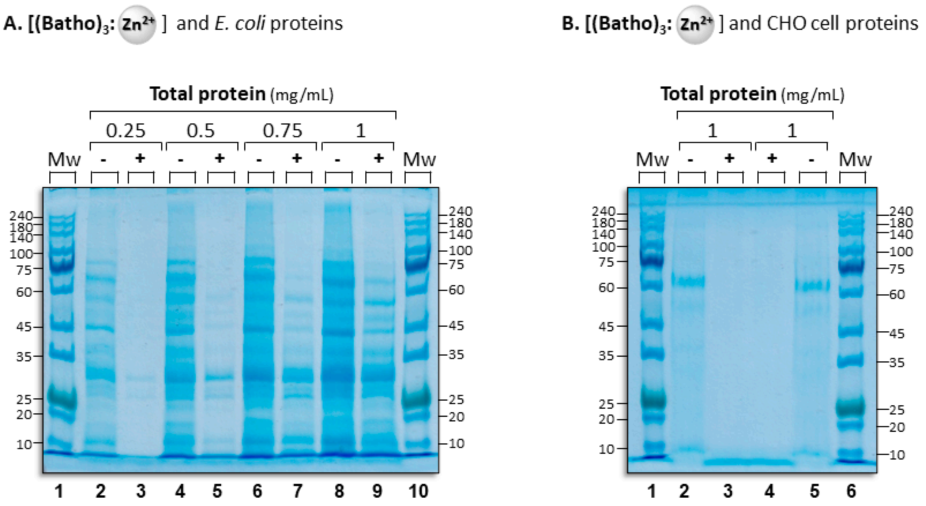

Figure S1: SDS-PAGE electrophoresis (with β-mercaptoethanol) displaying background impurity proteins present in the supernatant with (**+**) or without (**-**) the [(batho)$_3$:Zn^{2+}] complex. Total protein concentration was determined by the Bradford assay as described in the Experimental section. **A.** *E. coli* proteins; **B.** CHO cell proteins (in duplicate). Molecular weight markers are shown **(A)** in Lanes 1 and 10; **(B)** Lanes 1 and 6.

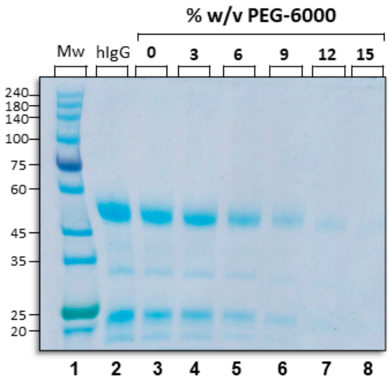

Figure S2: SDS-PAGE electrophoresis (with β-mercaptoethanol) of the supernatant reveals increasing capture of hIgG by the [(batho)$_3$:Zn^{2+}] complex as a function of PEG-6000 concentration. Lane 1: Molecular weight markers; Lane 2: total amount of hIgG added to each of the purification trials (15 mg/mL); Lanes 3-8: hIgG remaining in the supernatant following incubation with the [(batho)$_3$:Zn^{2+}] complex. Preparation of the complex and precipitation with NaCl were performed as described in the Materials and Methods section. The precipitated complex was resuspended in the presence of 15 mg/mL hIgG in 50 mM sodium phosphate (pH 7.0) with PEG-6000 concentration between 0 and 15 %w/vol in DDW. The suspension was vigorously vortexed for 2 minutes and incubated at 10°C for 30 minutes. A short spin was applied (21,000*xg*, 5 minutes at 10 °C) and the supernatant composition was analyzed by SDS-PAGE.
